# Assessment of disease activity by patients with juvenile idiopathic arthritis and the parents compared to the assessment by pediatric rheumatologists

**DOI:** 10.1186/1546-0096-11-48

**Published:** 2013-12-24

**Authors:** Wineke Armbrust, Jolanda G Kaak, Jelte Bouma, Otto T H M Lelieveld, Nico M Wulffraat, Pieter J J Sauer, Eric van Sonderen

**Affiliations:** 1 Department of Pediatric Rheumatology, University Medical Center Groningen, Beatrix Children’s Hospital, University of Groningen, Groningen, the Netherlands; 2 University Medical Center Groningen, Beatrix Children’s Hospital, University of Groningen, Groningen, the Netherlands; 3 Department of Health Sciences, University Medical Center Groningen, Health Psychology Section, University of Groningen, Groningen, the Netherlands; 4 Department of Rehabilitation,University Medical Center Groningen, University of Groningen, Groningen, the Netherlands; 5 Department of Childrens Rheumatology and Immunology, University Medical Center Utrecht, Wilhelmina Children’s Hospital, University of Utrecht, Utrecht, the Netherlands

**Keywords:** Juvenile idiopathic arthritis, Pain, Self care, Self report

## Abstract

**Background:**

Self assessment of arthritis is important for recognition of disease activity and early initiation of therapy. Proper interpretation of physical symptoms is necessary for this. The purpose was to investigate the assessment by patients and parents of disease activity in juvenile idiopathic arthritis (JIA) and to compare their assessments to rheumatologists’ assessments.

**Methods:**

Patients and parents assessed 69 joints on a paper homunculus and marked each joint with a different color according to presumed presence of disease: active disease (AD), doubt, and non-active disease (NAD). Their assessments were compared to the rheumatologists’ assessments. If patients and/or parents marked an inflamed joint, it counted as AD. Pain, functional impairment, and disease duration were analyzed to differentiate more precise between true and false positive and true and false negative assessments.

**Results:**

We collected assessments of 113 patients and/or parents. AD was assessed 54 times, 33 of which were true positives. NAD was assessed 23 times, 22 of which were true negatives. Doubt was expressed 36 times, 9 of which were assessed by the rheumatologist as AD. Sensitivity and specificity of AD was 0.77 and 0.31. Pain and functional impairment scored highest in AD, intermediate in doubt, and lowest in NAD.

**Conclusion:**

Patients and/or parents seldom missed arthritis but frequently overestimated disease activity. Pain, functional impairment, disease duration, gender, and age did not differentiate between true and false positives for. Patients perceived JIA as active if they experienced pain and functional impairment. To reduce overestimation of the presence of AD we need to improve their understanding of disease activity by teaching them to distinguish between primary symptoms of JIA and symptoms like pain and functional impairment.

## Background

Juvenile idiopathic arthritis (JIA) is a chronic autoimmune disease characterized by periods of active disease alternated by periods of remission. Forty to sixty percent of patients achieve remission and stay in remission without medication for varying lengths of time [1,2]. Disease activity in JIA can be determined by core set criteria [3,4] and the juvenile arthritis disease activity score [5,6]. These methods include parent and patient assessments on global disease activity, the erythrocyte sedimentation rate, assessments by pediatric rheumatologist of pain, limitation, and inflammation of all joints, and rating global disease activity [3-7]. Whether arthritis is actually present is a key issue in determining disease activity and initiating treatment.

Although disease activity is assessed regularly by a rheumatologist, early detection of disease activity at home, between scheduled consultations, is a major concern. According to current best practice patients with JIA should be treated as soon as symptoms appear. Treatment must be aimed at early remission so as to prevent long-term complications as joint damage and to improve prognosis [8-10]. Underestimating disease activity by patients and their parents invariably leads to delayed treatment with joint damage as a consequence. It is equally important not to overestimate disease activity. Overestimation may lead to the patient taking less part in sport and leisure activities, missing school, and excessive medication. The reduced levels of activity that result in the deterioration of physical fitness are a major concern in JIA patients. A previous study reported decreased physical fitness in JIA patients even during periods of remission [11,12]. It is necessary to stimulate active participation in sport and other activities, while at the same time fine-tuning these activities to disease activity. Early control of disease activity can lead to rapid remission and timely return to daily activities [11-14]. For these reasons it is important that we educate patients and parents to assess disease activity accurately.

The reliability of self-reported counts of swollen joint by adults with rheumatoid arthritis compared to the assessments by rheumatologists and/or ultrasonography is poor [15-18]. In children with JIA self-assessment of disease activity by the patients and/or their parents by indicating inflamed joints has not been investigated. A study on rating global disease activity using Visual Analogue Scales (VAS) showed discordance between parents’ and rheumatologists’ assessments. This was evident especially in cases where the patient had awarded high scores for pain and had indicated significant functional impairments or in cases where the rheumatologist had indicated disease activity in a large number of joints [19,20]. Correct assessment at home is important in order to report disease activity to the rheumatologist without delay and so preventing the patient from feeling more limited than necessary. Pinpointing arthritis to one particular joint may be difficult for patients and/or parents while, in fact, the important issue is to determine the presence of disease activity. Indicating disease activity in another joint than the one identified by the rheumatologist is not necessarily a wrong assessment, since it may still lead to early detection of disease activity. Correct assessment of disease activity in a particular joint is, therefore, less important than correct assessment of disease activity per se.

The aim of our study was to evaluate the assessment of disease activity in children with JIA by patients and/or their parents, and to determine the factors that influenced their assessments.

## Methods

### Participants

All patients aged 4 to18 years and their parents attending the outpatient clinics of the Beatrix Children’s Hospital, University Medical Center Groningen and the Wilhelmina Children’s Hospital, University Medical Center Utrecht between March and June 2010, and who were diagnosed with JIA according to the revised criteria of the International League of Associations for Rheumatology (ILAR) [21], were eligible subjects. Any patient not living with the parent that accompanied him or her on a daily basis was excluded, as were patients and/or parents not in command of Dutch or English. According to the Institutional Review Board of both hospitals was this study exempt from approval. Therefore no written informed consents were obtained from parents and/or children.

We collected information on patient characteristics such as age, gender, and disease duration. Patients were categorized according to the ILAR criteria [21]. Extended oligoarticular JIA, rheumatoid factor positive and rheumatoid factor negative, and polyarticular JIA were considered as one group.

**Figure 1:**
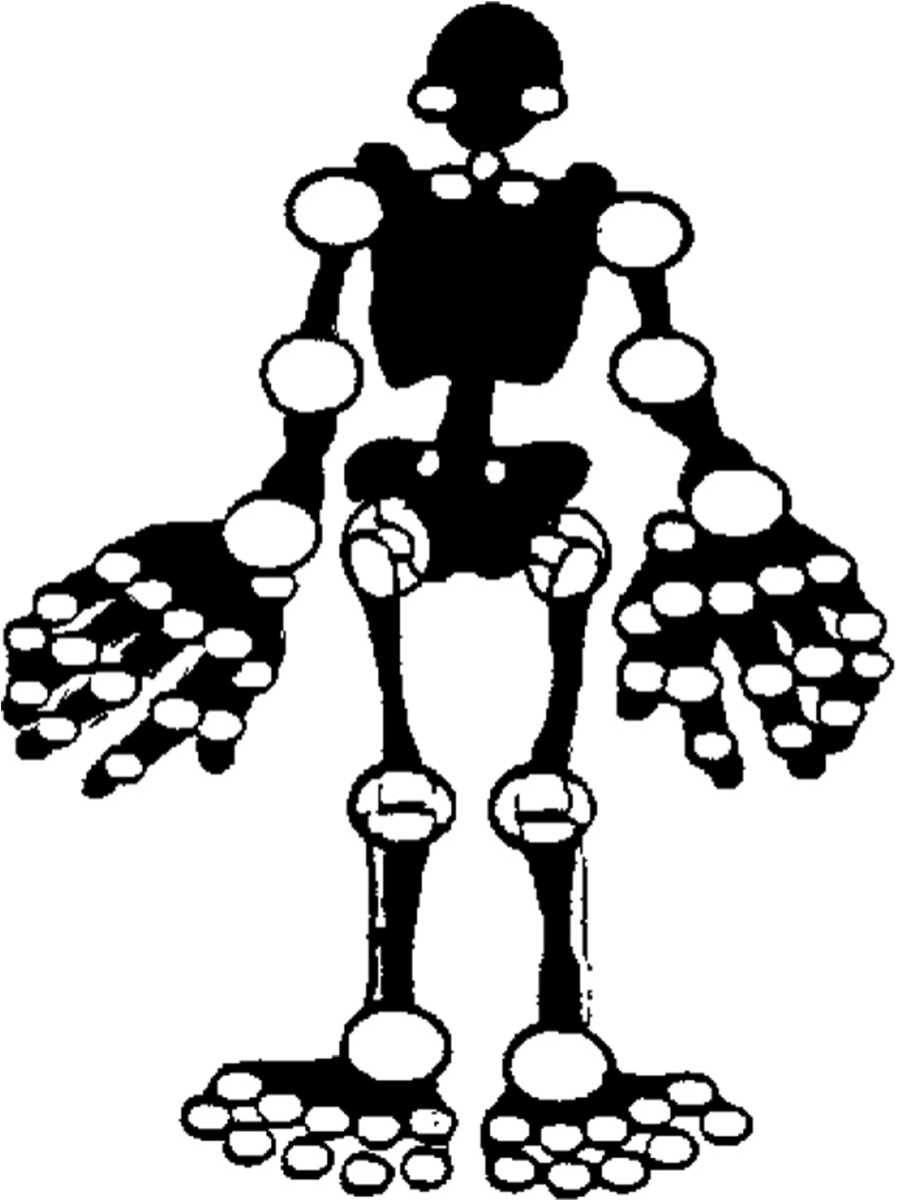
Homunculus.

Fifteen to thirty minutes prior to the visit to the pediatric rheumatologist (hereafter referred to as the rheumatologist), the patient and one or both parents were asked to *independently* assess 69 joints on a paper homunculus. Patients younger than nine years were assisted by an independent student, after this age both patient and parent each filled out an assessment form separately. The homunculus (Figure 1) displayed all joints, except those judged as too difficult to assess, i.e. the acromioclavicular joint, and the thoracic and lumbar joints of the spine. The ankle and wrist were scored as a collective joint. Patients were instructed to mark the joints with three different colors: in case arthritis was presumed active in a joint it was marked red, in case of doubt it was marked yellow, and in case no disease activity was perceived it was marked green. At the time JIA had been diagnosed, the symptoms of arthritis had been explained to the patient and their parents by their own rheumatologist as part of routine practice. Thus, when the homunculus was handed out no further instruction on how to identify arthritis was given.

After the rheumatologist had seen the patient he or she also marked the joints on the homunculus according to the same instruction when to use red, yellow, or green. The rheumatologist was blinded for the results of the patients and parents.

Prior to their visit to the rheumatologist patients were asked to complete the Dutch version of the Childhood Health Assessment Questionnaire (CHAQ) for the last seven days before the visit. It measures functional impairment in eight domains, i.e. getting up, dressing and grooming, eating, walking, hygiene, reaching, gripping, and activities [22]. Scores range from 0 to 3, where 0 stands for no impairment and 3 for maximum impairment. The patient and/or the parents also filled out the Visual Analogue Scale (VAS) to measure pain. It ranges from 0 to 100 mm on a linear scale, where zero stands for no pain and 100 for maximum pain. If the patients were younger than nine years the parents completed the CHAQ and the VAS with consulting the patients.

### Analysis

#### Disease activity

Disease activity was based on the overall joint assessments made by the patient, the parent, and the rheumatologist. The patients’ , parents’ , and rheumatologists’ assessments were divided into three categories: active disease (AD) if at least one joint was colored red, non-active disease (NAD) if all the joints were colored green, and doubt if at least one joint was colored yellow in the absence of any red joints.

### Patients’ and/or parents’ assessments compared to the rheumatologists’ assessments

The assessments by the patients and/or parents were compared to the assessments by the rheumatologists. The latter assessments were regarded as the criterion standard, since this is standard procedure when ultrasound examinations are not routinely performed [23,24]. The assessments were divided into six categories: true positive, false positive, true negative, false negative, doubt expressed by either patient or parent while the rheumatologist indicated AD, and doubt expressed by either patient or parent while the rheumatologist indicated NAD. In order to examine whether patients and parents together were better able to assess AD, their assessments were combined whereby a positive score awarded by either the parent or the child was considered as positive. The choice to analyze the combined assessments stemmed from our opinion, that the consequences of missing AD are more harmful than if it were overestimated. We examined the combined assessments in the same way as the separate ones. At home, in case of presumed disease activity, the decision of the patients or their parents, to either contact a rheumatologist or not, will generally be a joint conclusion arrived at by both the patients and their parents. Subsequent analyses were, therefore, performed with the combined assessments.

### Sensitivity and specificity

We calculated the sensitivity and specificity of the patients’ and parents’ assessments separately and of the combined assessments.

### Analysis of the variables that influenced parents’ and/or patients’ assessments

We analyzed whether the variables of functional ability (based on CHAQ), pain (based on VAS), gender, age, and disease duration influenced patients’ and/or parents’ opinion about the presence or absence of JIA, and whether these variables discriminated between AD and NAD as assessed by the rheumatologist.

## Results

One hundred and thirteen patients, whose main characteristics are presented in Table 1, were included together with at least one parent. None of the patients refused to participate. None of the patients had extra articular manifestations as fever rash or enthesitis.

**Table 1:** Patient characteristics

**Characteristic**	**Sample (%)**	**Median (range)**	**Mean (SD)**
	**n = 113**		
Gender			
- Male	37 (32.7)		
- Female	76 (67.3)		
Age (years)		11.4 (3.8)	12 (3-18)
- <9	27 (23.9)		
- 9-12	35 (31.0)		
- >12	51 (45.1)		
Condition			
- Oligoarthritis	43 (38.1)		
- Polyarthritis*	55 (48.7)		
- ERA^†^	4 (3.5)		
- Systemic JIA^#^	9 (8.0)		
- Other arthritis	2 (1.8)		
Disease duration (months)		59.1 (49.5)	48 (0-192)
- = < 12	22 (19.5)		
- = > 13	91 (80.5)		
CHAQ score		.41 (.52)	.13 (0-2.38)
- 0	45 (39.8)		
- = < 1	45 (39.8)		
- >1	19 (16.8)		
- Missing	4 (3.5)		

*Including extended oligo arthritis, ^†^Enthesitis related JIA, ^#^No extra-articular manifestations were present at time of the study.

### Disease activity

In Table 2 we show the results of the assessments by the patients, the parents, and the combination of patients and parents, compared to the assessments of the rheumatologists. Patients indicated AD in 50 cases, doubt in 34 cases, and NAD in 29 cases. Parents indicated AD in 41 cases, doubt in 43 cases, and NAD in 29 cases. The combination of patients’ and parents’ assessments shows AD in 54 cases, doubt in 36 cases, and NAD in 23 cases. Rheumatologists assessed AD in 43 patients and NAD in 70 patients and doubt was expressed three times. In all these last cases this resulted in adjusting treatment by, for example, advancing regular consultations or more detailed tests. Therefore, in those situations where the rheumatologists had expressed doubt, we considered it a case of AD.

**Table 2:** Patients’ and/or parents’ assessments compared to the rheumatologists’ assessments

		**Rheumatologist**		
		**AD**	**NAD**	**Total**
	AD	A	B	
Patient		31 (.72)^*^	19 (.27)	50 (.44)
Parent		30 (.70)^*^	11 (.16)	41 (.36)
Combination		33 (.77)^*^	21 (.30)	54 (.48)
	Doubt	C	D	
Patient		10 (.23)	24 (.34)	34 (.30)
Parent		12 (.28)	31 (.44)	43 (.38)
Combination		9 (.21)	27 (.39)	36 (.32)
	NAD	E	F	
		2 (.05)	27 (.39)^†^	29 (.26)
		1 (.02)	28 (.40)^†^	29 (.26)
		1 (.02)	22 (.31)^†^	23 (.20)
Total				
Patient		43 [1]	70 [1]	113 [1]
Parent		43 [1]	70 [1]	113 [1]
Combination		43 [1]	70 [1]	113 [1]

NAD = Non-active disease, AD = Active disease, A = True positive, B = False positive, C = Doubt in NAD, D = Doubt in AD, E = False negative, F = True negative, n(percentage).

^*^n (sensitivity), ^†^n (specificity).

For AD the sensitivity and specificity of the combined patient/parent assessments were 0.77 (cell A, true positives) and 0.31 (cell F true negatives), respectively. If doubt was interpreted as AD, sensitivity increased to 0.98 (cells A + C). But the specificity remained low at about 0.31 (cell F). These results indicate that AD was rarely missed, while it is overrated considerably.

Positive agreement, i.e. the agreement between patients and parents on the presumed presence of JIA, was 87%. These results confirmed our choice to perform further analyses with the combined assessments of parents and patients because we assumed that the decision taken at home to either contact the rheumatologist or not, will be arrived at by patient and parent together.

Our analysis of the factors that influenced the parents’ and patients’ assessment revealed interesting facts. In Table 3 we present the results of the patient characteristics, the pain scores, and functional abilities in relation to the presence or absence of disease activity. Age of the patients, gender, and duration of disease did not differ between cases with and without AD. The pain scores, as indicated by patients and/or parents as well as scores of functional impairment, were highest in the category in which patients and/or parents scored AD (cells A + B). Within this category pain scores and functional impairment were not different between the true positive and false positive assessments. Pain scores and functional impairment were intermediate in the category where patients and/or parents expressed doubt (cells C + D). There were no differences in this group between AD or NAD as assessed by the rheumatologists. Functional impairment scores approaching zero were seen in assessments in which NAD was rated by the patients and/or parents irrespective of whether this assessment was a true negative or a false negative (cells E + F).

**Table 3:** Patient characteristics by assessment category

**Combination**		**Rheumatologist**	
**Parent/patient**		**AD**	**NAD**
	AD	A	B
		N =33	N =21
Age		11.2 (4.2)	11.2 (3.7)
Gender (M:F)		12:21	4:17
VAS patient		33.5 (28.0)	27.9 (30.1)
VAS parent		31.6 (25.5)	38.5 (30.9)
CHAQ		0.7 (0.6)	0.6 (0.4)
Dis.duration		53.1 (52.9)	60.1 (49.5)
	Doubt	C	D
		N =9	N =27
Age		11.8 (3.2)	12.2 (3.6)
Gender (M:F)		2:7	9:18
VAS patient		19.0 (27.0)	17.0 (19.9)
VAS parent		20.4 (25.1)	18,2 (23.8)
CHAQ		0.3 (0.5)	0.3 (0.4)
Dis. duration		54.2 (39.3)	77.3 (51.9)
	NAD	E	F
		N =1	N =22
Age		6.0	11.1(3.8)
Gender (M:F)		M = 1	9:13
VAS patient		20 (0)	0,9 (1.6)
VAS parent		0.0 (0)	0.9 (2.0)
CHAQ		0 (0)	0.04 (0.1)
Dis.duration		44	47.4 (43.8)

NAD = Non-active disease, AD = Active disease, A = True positive, B = False positive, C = Doubt in NAD, D = Doubt in AD, E = False negative, F = True negative.

Age in years, M = male, F = female, VAS pain patient and parent 0-100 mm, Dis duration = disease duration in months.

Mean (SD).

## Discussion

This is the first study in children with JIA that compared the assessment of disease activity by color-coding the joints displayed on a homunculus by patients and/or parents to the assessments of rheumatologists. We found that patients and/or parents more frequently presumed disease activity to be present. In only one case had the rheumatologist indicated disease activity that had not been indicated by the patients and/or parents. Parents and patients agreed strongly on the presence or absence of disease activity. For patients pain and functional impairment were important determinants in the assessment of active disease, but these variables did not discriminate between correct and false assessments.

There are several reasons why it is important that patients and parents are able to accurately assess the current state of JIA. If they are able to detect disease activity correctly, it will be treated as soon as it is recognized so as to achieve early remission and to prevent long-term damage [8]. If parents and patients are able to assess disease activity adequately, regular consultations can be adjusted accordingly. If patients underestimate disease activity adequate medical treatment may be delayed and they may exceed their physical limits, the consequences of which are unknown. Finally, if patients are to overestimate disease activity this may lead to their taking part less in sport and leisure activities. This is an undesirable state of affairs because the already existing reduced level of physical fitness and exercise capacity of patients with JIA may be reduced even further [11-13].

We found that patients and/or parents overestimated disease activity more frequent than that they missed disease activity. A considerable number of patients and/or parents expressed doubt about disease activity. Taking into consideration the fact that the consequences of missing active disease are more harmful than overestimating disease activity, patients and parents who express doubt should be advised to consult their rheumatologist. Our results show that if consultations are to be regulated on the basis of patients’ and/or parents’ assessments, barely any case of active disease will be missed, but it will lead to many unnecessary visits. During such visits, however, patients can be reassured that the disease is currently not active and the patients could be stimulated to continue or increase their normal daily activities and sport.

Why parents and patients overestimated disease activity is an interesting question. One explanation could be that parents and patients are more afraid of missing disease activity than of overestimating it. Most patients had a long history before JIA was diagnosed during which symptoms were underestimated or misinterpreted, and this has a marked psychological impact [25,26]. Secondly, parents and patients are aware that delaying treatment when the disease is active could be harmful, thus rather overestimate disease activity just to be at the safe side.

In this study we took the rheumatologist’s assessment as the criterion standard, which is common practice. Current disease activity scores are based on clinical and laboratory parameters combined with rheumatologists’ assessments [3-6]. Laboratory parameters were not included in this study while pure clinical parameters were compared. One could, however, question the reliability of a rheumatologist’s joint assessments. Good interobserver reliability between rheumatologists of articular assessment in children with JIA was reported [27]. More recent publications, however, showed that the presence and absence of arthritis as assessed by the rheumatologist or the patient is not always confirmed by ultrasound [17,28-31]. That rheumatologists sometimes miss disease activity could possibly be an explanation for the overestimation of disease activity by patients and/or parents as we found in our study. The value of ultrasound and MRI to monitor disease activity in patients with JIA seems promising but has yet to be investigated in more detail [31-34]. We found that patients who indicated active disease and those who expressed doubt both had high pain scores and experienced functional impairment in executing their daily activities. Analysis of functional impairment, pain, and disease duration in the patients did not differentiate between those with AD as assessed by the rheumatologist and those without AD. Functional impairment, pain, and disease duration also did not differentiate between true positive and false positive assessments of the parents and/or patients. This suggested that parents’ and/or patients’ perception of disease activity was based on pain and impairment.

This finding confirmed the work by Consolaro et al., who studied agreement on disease activity ratings between parents and rheumatologists as measured by global assessment scores [19]. They also found that parents tended to award higher disease activity scores compared to the rheumatologist if their child felt pain or was impaired. It makes no difference whether patients and parents were asked to fill out a global assessment or whether they had to make a more precise joint count on a homunculus as we required in our study. A recent study on adult patients with rheumatoid arthritis showed that the most significant determinants for discrepancies between the patients’ and the rheumatologists’ assessments of global disease activity scores are pain and joint swelling [35]. Adult patients’ assessments of disease activity by means of a joint count on a homunculus compared to those of rheumatologists’ appears to be unreliable [16].

Pain is a major problem in children with JIA and it is not always related to disease activity or damage. It can be caused by pathophysiological and psycho-emotional factors [36-42]. Pain is related to well-being and should be a major concern in the treatment of children with JIA [43]. In our opinion, monitoring pain and well-being are important in the management of JIA and it needs the attention of the clinicians, but we question whether these subjective patient-related factors should be included in the assessment of disease activity.

We need to teach patients and their parents to recognize the symptoms of JIA and how to recognize current disease activity. This is necessary so medical treatment can be initiated promptly in case of AD and normal activities can be continued or resumed in case JIA is not active. If the patient perceives the disease as being active while this is not confirmed by the rheumatologist, the reasons on which the patient bases his or her assessment should be discussed. If pain and functional impairment are the patient’s main reasons for presuming the presence of JIA, while it is not confirmed by the rheumatologist, more detailed tests are needed to exclude whether local deconditioning, damage, or emotional factors are involved.

True AD requires adjustment of medication, while other factors leading to AD being perceived requires education and proper counseling. Efficient self-management is important if patients are to cope with a chronic disease [44-46]. It is important, therefore, that children with JIA learn to recognize the symptoms and that they are treated correctly.

We identify some limitations of this study. We did not take into account whether a joint that had been affected in the past was more frequently marked as being inflamed again. As a consequence, we were unable to determine whether arthritis in the past had influenced current assessment. Another limitation was that we did not ask on what grounds a patient and/or a parent considered the disease to be active in a joint. Thirdly, we did not identify morning stiffness what was added in the latest criteria of remission [47]. Morning stiffness could be a factor that elevates the positive predictive value of the assessments of the patients and parents. Finally we did not use ultrasound to verify the rheumatologists’ assessments.

## Conclusion

In this study we found that patients and parents barely missed arthritis while overestimation occurred frequently. The perceived presence of arthritis was related to the presence of pain and functional impairment. In order to reduce the frequency of over-reporting active disease, we need to educate both patients and their parents to distinguish between pain, impairment and disease activity, and to recognize the presence of active disease unerringly.

## Competing interests

The authors declared that they have no competing interests.

## Authors’ contributions

WA carried out the study design and conception, data collection, data analysis, data interpretation, and writing of the manuscript. JK carried out study design and conception, data collection, data analysis, data interpretation, and revising of the manuscript. JB carried out study design and conception, data interpretation and revising of the manuscript. OL carried out study design and conception, data interpretation and revising of the manuscript. NW carried out study design and conception, data collection and revising of the manuscript. PS carried out study design and conception, data interpretation and revising of the manuscript. ES carried out study design and conception, data analysis, data interpretation and revising of the manuscript. All authors read and approved the final manuscript.
